# Supplementary material for: On the Photostability of Cyanuric Acid and Its Candidature as a Prebiotic Nucleobase
Source: Molecules. 2022 Feb 10;27(4):1184. doi: 10.3390/molecules27041184 (PMC8875432; doi:10.3390/molecules27041184)
Supplement: Supplementary file 1 [file molecules-27-01184-s001.zip › molecules-1538829-supplementary.pdf]

On the Photostability of Cyanuric Acid and its Candidature as a Prebiotic Nucleobase

Luis A. Ortiz-Rodríguez,[1,*] Sean J. Hoehn[1] and Carlos E. Crespo-Hernández[1,*]

[1]Department of Chemistry, Case Western Reserve University, Cleveland, OH, 44106.

* Correspondence: lao21@case.edu, carlos.crespo@case.edu

Electronic Supporting Information

I. Supporting Results

Table S1. Vertical excitation energies of microsolvated cyanuric acid (CA) calculated for the first six excited singlet states at the TD-X/CPCM/def2-TZVP in water, where X is the functional used. Oscillator strengths are shown in parentheses.

State	ωB97X	LC-PBE
S_1(nπ*)	6.46 (0.000)	6.56 (0.000)
S_2(nπ*)	6.46 (0.000)	6.56 (0.000)
S_3(nπ*)	6.96 (0.000)	7.02 (0.000)
S_4(ππ*)	7.09 (0.000)	7.35 (0.000)
S_5(ππ*)	7.85 (0.88)	7.99 (0.92)
S_6(ππ*)	7.85 (0.88)	7.99 (0.92)

Table S1 shows the results obtained for the vertical excitation energies of microsolvated CA using the range-separated hybrid functionals ωB97X[1] and LC-PBE.[2] As can be seen in Table S1, the vertical energies are within the mean deviation error (0.2-0.3 eV) of vertical excitation energies obtained with electronic-structure methods.[3,4] They are also within the error of the vertical excitation energies obtained with the CAM-B3LYP functional (see main text, Table 1).

Table S2. Vertical excitation energies for the lowest twelve singlet and twelve triplet states of microsolvated CA calculated at the TD-PBE0/C-PCM/def2-TZVP//X3LYP/C-PCM/cc-pVDZ level of theory in water.

State	Energy (eV)
S_1 (nπ*)	6.11 (0.000)
S_2 (nπ*)	6.11 (0.000)
S_3 (ππ*)	6.68 (0.000)
S_4 (nπ*)	6.71 (0.000)
S_5 (84% nπ*+ 16% ππ*)[a]	7.33 (0.020)
S_6 (nπ*)	7.34 (0.006)
S_7 (ππ*)	7.36 (0.104)
S_8 (ππ*)	7.36 (0.104)
S_9 (nπ*)	7.54 (0.001)
S_{10} (ππ*)	7.64 (0.000)
S_{11} (ππ*)	7.76 (0.72)
S_{12} (ππ*)	7.76 (0.72)
T_1 (nπ*)	5.74
T_2 (nπ*)	5.74
T_3 (ππ*)	5.76
T_4 (ππ*)	5.76
T_5 (ππ*)	5.81
T_6 (nπ*)	6.32
T_7 (ππ*)	6.49
T_8 (ππ*)	6.69
T_9 (ππ*)	6.70
T_{10} (nπ*)	7.28
T_{11} (nπ*)	7.28
T_{12} (nπ*)	7.48

[a] This is the only state that has significant contribution of more than one character.

The vertical excitation energies of microsolvated CA for the lowest twelve singlet and triplet states with their respective character calculated at the TD-PBE0/C-PCM/def2-TZVP//X3LYP/C-PCM/cc-pVDZ level of theory in water are reported in Table S2. As can be observed in Table S2, only the S_6 (ππ*), S_7 (ππ*), S_{11} (ππ*) and S_{12} (ππ*) excited singlet states have oscillator strengths that are large enough to have a significant contribution in the absorption spectrum of CA and thus, in principle should be the only states that could be accessed upon direct excitation of CA. We highlight that these results are in contrast with the results obtained with the range separated hybrid functionals (i.e., CAM-B3LYP, ωB97X and LC-PBE, Table 1 and Table S1) where only the S_5 (ππ*) and S_6 (ππ*) states should be populated. They are included here to primarily highlight this discrepancy between the used DFT functionals. We are hopeful that the discrepancies found between the CAM-B3LYP, ωB97X, and LC-PBE and the PBE0 functional will motivate new investigations of this system using multiconfigurational approaches.

Regardless, and for completeness, it may be informative to discuss the relaxation pathways predicted using the TD-PBE0/C-PCM/def2-TZVP//X3LYP/C-PCM/cc-pVDZ level of theory. Using the El-Sayed's propensity rules[5,6] and a 0.5 eV energy gap threshold as selection criteria,

the following transitions to the triplet manifold (following internal conversion from higher- to lower-lying excited singlet states) are predicted: S_{12}-T_{12}, S_{11}-T_{12}, S_{10}-T_{12}, S_{10}-T_{11}, S_{10}-T_{10}, S_8-T_{11}, S_8-T_{10}, S_7-T_{11}, S_7-T_{10}, S_4-T_9, S_4-T_8, S_4-T_7, S_3-T_6, S_2-T_4, S_2-T_3, S_1-T_4 and S_1-T_3. As described in the main text using the CAM-B3LYP results, we calculated spin-orbit couplings between these transitions (Table S3). As shown in Table S3, there are transitions with spin-orbit couplings that are relatively large (>10 cm^{-1}), which suggest that if CA is photoactivated and populates the S_6(ππ*), S_7(ππ*), S_{11}(ππ*) and S_{12}(ππ*) excited singlet states, intersystem crossing to the triplet manifold may compete with internal conversion to the S_1 and subsequent internal conversion to the ground-state through a S_1/S_0 conical intersection. Therefore, the results obtained with the PBE0 functional suggest that CA should also be photostable under the solar radiation conditions that reached the Earth surface during the prebiotic era but that the probability of photochemical damage somewhat increases when CA is exposed to radiation wavelengths shorter than ca. 200 nm due to the slightly higher probability of intersystem crossing.

Table S3. Energy gaps between the calculated excited singlet and triplet states and their corresponding spin-orbit couplings obtained for microsolvated CA at the TD-PBE0/C-PCM/def2-TZVP//X3LYP/C-PCM/cc-pVDZ in water.

Transition	ΔE (eV)	SOCs (cm^{-1})
S_1-T_3	0.35	17
S_1-T_4	0.35	17
S_1-T_5	0.30	16
S_2-T_3	0.35	17
S_2-T_4	0.35	17
S_2-T_5	0.30	16
S_3-T_6	0.36	0.27
S_4-T_7	0.22	0.13
S_4-T_8	0.02	6.7
S_4-T_9	0.01	6.7
S_7-T_{10}	0.08	15
S_7-T_{11}	0.08	15
S_8-T_{10}	0.08	15
S_8-T_{11}	0.08	15
S_{10}-T_{10}	0.36	7.0
S_{10}-T_{11}	0.36	7.0
S_{10}-T_{12}	0.16	0.34
S_{11}-T_{12}	0.28	13
S_{12}-T_{12}	0.28	13

II. Supporting References

(1) Chai, J.-D.; Head-Gordon, M. Systematic Optimization of Long-Range Corrected Hybrid Density Functionals. *J. Chem. Phys.* **2008**, *128* (8), 84106.

(2) Iikura, H.; Tsuneda, T.; Yanai, T.; Hirao, K. A Long-Range Correction Scheme for Generalized-Gradient-Approximation Exchange Functionals. *J. Chem. Phys.* **2001**, *115* (8), 3540–3544.

(3) Silva-Junior, M. R.; Schreiber, M.; Sauer, S. P. A.; Thiel, W. Benchmarks for Electronically Excited States: Time-Dependent Density Functional Theory and Density Functional Theory Based Multireference Configuration Interaction. *J. Chem. Phys.* **2008**, *129* (10), 104103.

(4) Winter, N. O. C.; Graf, N. K.; Leutwyler, S.; Hättig, C. Benchmarks for 0--0 Transitions of Aromatic Organic Molecules: DFT/B3LYP, ADC (2), CC2, SOS-CC2 and SCS-CC2 Compared to High-Resolution Gas-Phase Data. *Phys. Chem. Chem. Phys.* **2013**, *15* (18), 6623–6630.

(5) El-Sayed, M. A. The Triplet State: Its Radiative and Nonradiative Properties. *Acc. Chem. Res.* **1968**, *1*, 8–16.

(6) El-Sayed, M. A. Spin-Orbit Coupling and the Radiationless Processes in Nitrogen Heterocyclics. *J. Chem. Phys.* **1963**, *38*, 2834–2838.
